# A case of squamous cell carcinoma of lung presenting with paraneoplastic type of acanthosis nigricans

**DOI:** 10.4103/0970-2113.76305

**Published:** 2011

**Authors:** Subhasis Mukherjee, Sudipta Pandit, Jaydip Deb, Arunabha Dattachaudhuri, Sourin Bhuniya, Pulakesh Bhanja

**Affiliations:** *Department of Respiratory Medicine, R.G. Kar Medical College and Hospital, Kolkata - 700 004, West Bengal, India*

**Keywords:** Acanthosis nigricans, lung cancer, non-small cell carcinoma, paraneoplastic syndromes

## Abstract

A 70-years-old male presented with blackening of both hands and face for last six months which was progressive and attended dermatology outpatients department. Dermatologist opined the skin lesions as acanthosis nigricans. He was referred to our department to evaluate for any underlying internal malignancy as he was a smoker. His chest X-ray revealed right sided hilar prominence with a mid zone cavity with fluid level. Fibreoptic bronchoscopy was done, there was one ulcerative growth in right middle lobe bronchus. Biopsy from the ulcer revealed probable squamous cell carcinoma. CT scan of thorax was also done and CT guided FNAC of Rt lung lesion yielded non small cell carcinoma. His skin lesions were also biopsied and diagnosis of acanthosis nigricans was confirmed. Here we report a case of acanthosis nigricans associated with non-small cell cancer of lung.

## INTRODUCTION

Acanthosis nigricans is a skin condition characterized by dark, thick, velvety skin in body folds and creases like typically in armpits, groin and neck. Sometimes the lips, palms or soles of the feet are affected as well. The skin changes appear slowly, sometimes over months or years. Rarely, the affected areas may itch. Acanthosis nigricans can begin at any age. Diagnosis is mainly clinical. Skin biopsy may confirm the diagnosis. Acanthosis nigricans is often associated with conditions that increase insulin level, such as type 2 diabetes or being overweight. In some cases, acanthosis nigricans is inherited. Certain medications, such as human growth hormone, oral contraceptives and large doses of niacin, can contribute to the condition. Rarely, acanthosis nigricans is associated with certain types of cancer; but interestingly adult onset acanthosis nigricans are almost always paraneoplastic, associated with internal malignancies.[1] There is no specific treatment for acanthosis nigricans.

## CASE REPORT

A 70-years-old male patient was apparently well six months back after which he noticed gradual blackening of dorsum of both hands and face since last six months. He attended dermatology outpatients department. Dermatologist clinically diagnosed the condition as acanthosis nigricans. He was referred to us for evaluation of any internal malignancy. On systematic questioning, the patient told that he had chronic cough with scanty expectoration for last one year which he ignored as smoker’s cough. There was history of three to four episodes of streaky hemoptysis in last one year. He also lost weight significantly in last six months though it was not documented. He was a heavy smoker with 30 pack-years of smoking. He also gave history of low-grade fever occasionally during this period but temperature was not recorded. He had no chest pain, shortness of breath. For these symptoms, he was taking homeopathic medicines without relief. There was one episode of epistaxis 12 months ago. There was no history of hematemesis, vomiting or convulsion.

**Figure 1:**
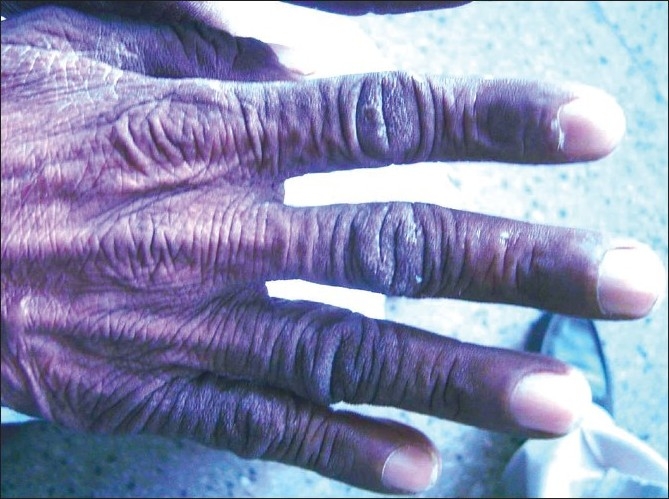
Hyperpigmented lesions of acanthosis nigricans

**Figure 2:**
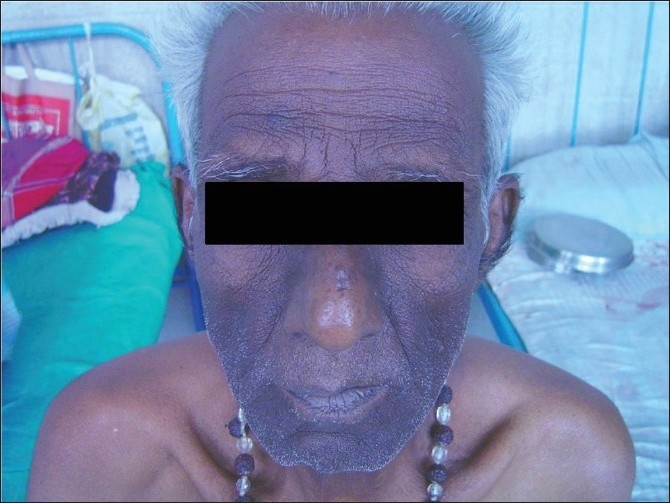
Hyperpigmented lesions of acanthosis nigricans over face

On general survey, his general condition was poor. He had moderate degree pallor and grade II clubbing, pulse rate was 92/min, blood pressure was 110/ 70 mm Hg. There was no palpable lymph node externally. There were hyperpigmented, velvety, rough and rugouse, papillomatous skin lesions symmetrically distributed over arms, armpit, face, neck and hands [Figures 1 and 2]. Examination of respiratory system revealed bronchial breath sound over right mammary area with crepitation over right mammary and axillary area. Abdomen was soft with no hepato-splenomegaly. Other systemic examinations were within normal limits.

Routine investigation revealed: hemoglobin-10.25 gm%, total leucocyte count was14000/mm^3^ with differential count being neutrophil-80%, lymphocyte-18%, eosinophil-01%, monocyte-01%. Blood sugar, serum urea, creatinine and liver function tests were all within normal limits. Sputum for Z-N staining (Ziehl-Neelsen) was negative on three occasions.

**Figure 3:**
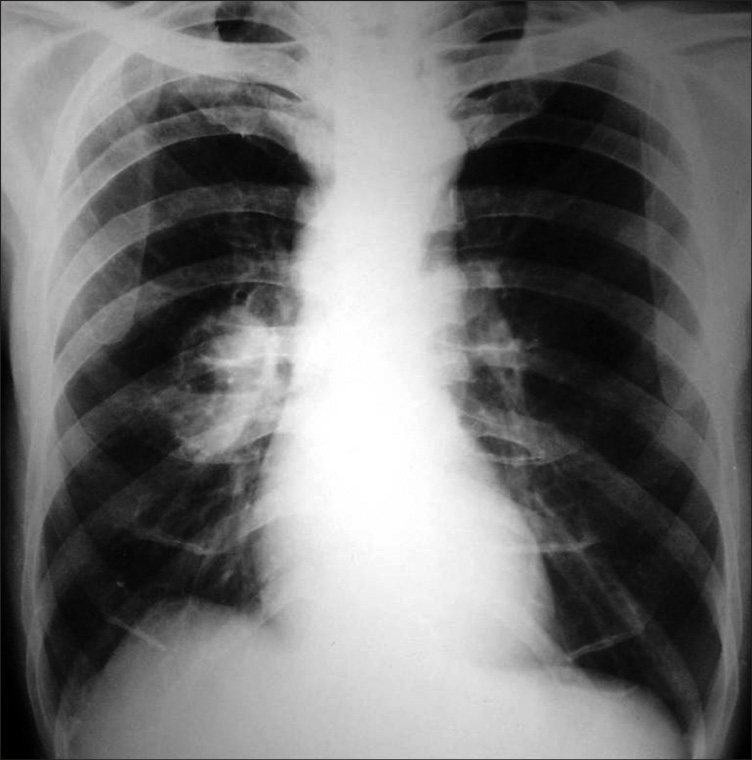
Chest x-ray showing right parahilar cavitary lesion

**Figure 4:**
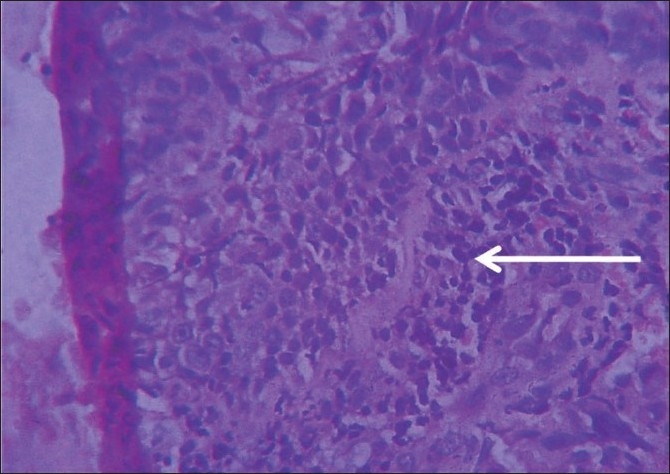
Histopathological slide of bronchial biopsy under high power field showing clusters of malignant squamous epithelial cells

**Figure 5:**
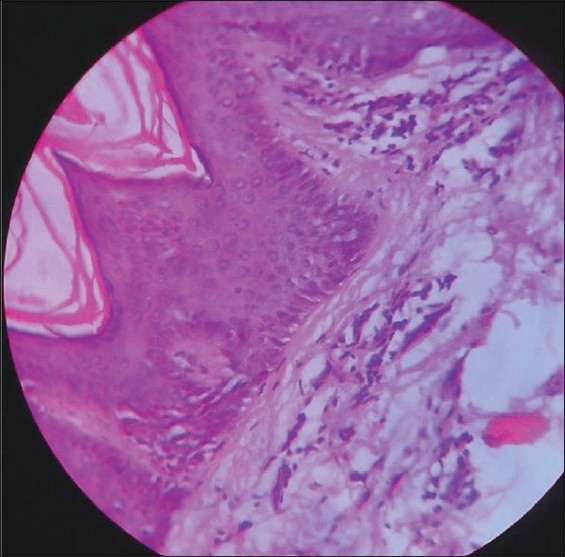
Histopathological slide of skin lesion under high power field showing features of acanthosis nigricans

Skiagram of chest (postero-anterior and right lateral view) revealed right hilar prominence and a thick walled irregular cavity around 3 cm diameter containing air fluid levels in the perihilar region occupying the area of right middle lobe[Figure 3]. Fibreoptic bronchoscopy was performed subsequently which revealed ulcerative growth in right middle lobe bronchus. Biopsy from the ulcer revealed squamous cell carcinoma[Figure 4]. Bronchial brushing and BAL(bronchoalveolar lavage) fluid culture for AFB(Acid fast bacillus) by BACTEC method were negative. CT scan of thorax with contrast and CT guided FNAC from the right middle lobe thick walled cavity was also done. CT guided FNAC of right lung lesion also yielded non small cell carcinoma. Ultrasonography of whole abdomen revealed no abnormality. Subsequently, biopsy was taken from skin lesion over right arm and it confirmed the skin lesions to be acanthosis nigricans[Figure 5].

## DISCUSSION

Various skin conditions are associated with some form of internal malignancy as paraneoplastic syndrome. These conditions are thought to be caused by aberrant behavior of white blood cells and their antibodies reacting either to the tumor or to substances produced by the tumor. Dermatomyositis, acanthosis nigricans, erythema multiforme, urticaria, scleroderma, hyperpigmentation, tripe palms are among the paraneoplastic skin lesions reported with various malignancies.[2] Acanthosis nigricans is a skin disorder characterized by darkening and thickening (hyperkeratosis) of the skin, occurring mainly in the folds of the skin in the armpit (axilla), groin and back of the neck. Acanthosis nigricans is not a skin disease per se but a cutaneous sign of an underlying condition or disease. There are two important types of acanthosis: benign and malignant. Although classically described as a sign of internal malignancy, this is very rare. Benign types, sometimes described as ‘pseudoacanthosis nigricans,’ are much more common.[2] An associated malignancy has been reported in over 90% of patients with tripe palms.[3] It is possible that tripe palms is merely the palmar manifestation of acanthosis nigricans. In one study malignancy-associated tripe palms was detected in 79 patients who had a total of 86 cancers. Fifty-seven of these patients also had acanthosis nigricans. Benign and malignant acanthosis nigricans are indistinguishable on histopathology. Involvement of mucous membrane is rare in benign variety. Adult onset acanthosis nigricans are associated with internal malignancies in almost 100% cases, these usually have sudden onset, rapid progression, profound hyperkeratosis and hyperpigmentation with coexisting pruritus.[4] In our case, age of the patient, rapid progression of skin lesions and involvement of oral mucous membrane were the points that hinted toward the presence of an underlying malignancy. Malignant acanthosis nigricans has been most commonly reported in association with tumors of gut (90%) especially with carcinoma of stomach,[5] liver, gall bladder and bile duct cancer,[6,7] and carcinoma of colon. Less frequently, it is associated with bronchogenic carcinoma,[8–10] breast cancer, ovarian cancer.[11] In 25-50% of cases, lesions are present in the mouth on the tongue and lips. If malignant acanthosis nigricans is suspected in a patient without known cancer, it is extremely important to perform a thorough workup for underlying malignancy and identify a hidden tumor. Acanthosis nigricans has no specific treatment, with treatment of primary disease, the condition may resolve. Corticosteroids and azathioprine have been tried in some patients with varying results. In our case, the patient was advised cytotoxic chemotherapy but he refused chemotherapy.

## CONCLUSION

Paraneoplastic acanthosis nigricans in patients with bronchogenic carcinoma is not very commonly reported in literature.
